# Electrical, Mechanical, and Thermal Properties of LDPE Graphene Nanoplatelets Composites Produced by Means of Melt Extrusion Process

**DOI:** 10.3390/polym9010011

**Published:** 2017-01-04

**Authors:** Karolina Gaska, Xiangdong Xu, Stanislaw Gubanski, Roland Kádár

**Affiliations:** Department of Materials and Manufacturing Technology, Chalmers University of Technology, SE 412-96 Gothenburg, Sweden; xiangdong.xu@chalmers.se (X.X.); stanislaw.gubanski@chalmers.se (S.G.); roland.kadar@chalmers.se (R.K.)

**Keywords:** graphene nanocomposites, low density polyethylene, field grading materials, electrical conductivity, dielectric response, mechanical properties, thermal properties

## Abstract

Composites of LDPE filled with different amounts of graphene nanoplatelets (GnP) were prepared in form of films by means of precoating technique and single screw melt-extrusion using two types of screws, compression and mixing. This manufacturing process imposes strong anisotropy on the sample’s morphology, in which the nanoplatelets become oriented along the extrusion direction. Such orientation of GnP in LDPE matrix is confirmed by scanning electron microscopy observations and it yields unique electrical properties. As compared to pure LDPE, significant reductions of the through-plane conductivity are found for the composites at relatively low electric fields (<20 kV/mm) at low filler concentrations. Above the field level of 20 kV/mm, a crossover effect is observed that results in a strong field dependency of the conductivity where the non-linear behavior starts to dominate. Moreover, differential scanning calorimetry (DSC) results indicate a decrease in polymer crystallinity of the composite matrix with increasing filler content, whereas thermogravimetric (TG) analysis shows a slight increase in the material’s thermal stability. Application of GnP also leads to improvement of mechanical properties, manifested by the increase of Young’s modulus and tensile strength in both types of samples.

## 1. Introduction

The world is in urgent need of a modernized power grid to meet the growing demands for a reliable, cost effective, and environmentally responsible power solution. The high voltage direct current (HVDC) technology is in this respect considered as the most feasible technical and economic solution [1] where extruded polymeric high voltage cables will be widely used for electric energy transportation across seas and inland. It is expected that the HVDC cables should be able to withstand extremely high electric stresses. However, as the weakest points of the cable technology are still located in terminations and joints [2,3], a precise control of electric field distribution in these components is of a crucial importance for maintaining their reliable operation [4]. This is presently achieved by applications of field grading materials consisting of polymer composites filled with semi-conducting particles—such as SiC, ZnO, or carbon black [2,3]—and the desired nonlinear electrical conductivity is provided by percolating filler. As such characteristics appear in composites with relatively high filler loading contents, 30–40 wt %, their mechanical properties are negatively affected as well as manufacturing processes become more demanding due to increased viscosity and tool wear. The alternative approach is to use conductive particles of graphene for the sake of its unique electrical, thermal, and mechanical properties [5,6]. The main advantage is the possibility of obtaining percolation thresholds at much lower loading contents [7,8,9], causing graphene-based nanocomposites to attract significant scientific interest [10]. The macroscopic properties of polymer nanocomposites are mainly dependent on the interfacial compatibility of polymer and filler particles, as well as polarity match between the graphene flake surfaces and the polymer [11]. Therefore, a proper dispersion and uniform distribution are the crucial issues. However, several studies indicated that the dispersion of graphene particles in polyolefin matrices become challenging due to the interfacial incompatibility of the constituents [12] and for this reason, selection of a proper manufacturing technique is essential. There have been a number of studies discussing methods of the composite fabrication and their influence on further materials properties [13]. For example, Kim et al., reviewed methods of dispersion into polymers and summarized resulting thermal, mechanical, and electrical properties of polymers filled with thermally reduced graphite oxide [14]. Kalaitzidou et al. [15,16] also compared potentially useful methods, such as melt compounding, solution intercalation, and in situ polymerization. Also, pre-coating compounding method, applied earlier by Drzal’s group [8,17,18] has shown that GnP nanocomposites can be produced with enhanced thermal and electrical properties.

This paper focuses on electrical, mechanical, and thermal properties of nanocomposites based on low density polyethylene (LDPE) filled with graphene nanoplatelets (GnP) and produced through the industrially attractive extrusion process. The influence of the extrusion parameters on filler dispersion and composites morphology are elucidated.

## 2. Materials and Methods

### 2.1. Materials

LDPE in the form of pellets was provided by Borealis AB (Stenungsund, Sweden). Graphene nanoplatelets originated from XG Sciences (xGnP M5 nanopowder, Lansing, MI, USA). Table 1 shows the properties of these components, in which the data for graphene nanoplatelets are as per the product data sheet, whereas the parameters of LDPE were measured by means of Gel Permeation Chromatography and Differential Scanning Calorimetry (DSC, Mettler Toledo Inc., Greifensee, Switzerland).

### 2.2. Materials Processing

#### 2.2.1. Precoating Technique

**Figure 1:**
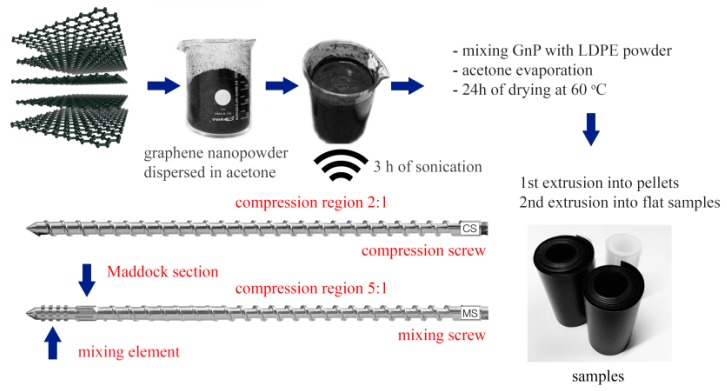
Scheme illustrating samples preparation process.

The manufacturing process of the studied specimens is illustrated in Figure 1. It follows the precoating technique used by Drzal et al. [16], where exfoliated graphene nanoplatelets coat LDPE powder. This technique is used in order to secure a good dispersion of graphene nanoplatelets during melt extrusion process.

Firstly, LDPE pellets were cryogenically grounded into powder with an average diameter of the particles of 0.5 mm. At the same time, GnP nanoplatelet powder was dispersed and exfoliated in acetone in a sonication bath for 3 h (90 W). Mixing of LDPE powder with exfoliated GnP was also performed in acetone, using an overhead stirrer rotating at 500 rpm for 40 min until full evaporation of acetone. Such obtained masterbatches were dried in an oven at 60 °C for 24 h.

#### 2.2.2. Melt Extrusion and Film Casting

GnP-LDPE masterbatches were extruded by means of a Brabender 19/25D (with a screw diameter *D* = 19 mm and a screw length of 25 D, Brabender GmbH & Co, Duisburg, Germany) single-screw extruder, equipped with a conveyor belt. Two types of screws were used during the extrusion: a compression screw (CS, compression ratio 2:1) and a mixing screw (MS, compression ratio 5:1) (see Figure 1). Compression screw provides a distributive mixing, based on continuous rearrangement of composite constituents, which secures high homogeneity of the extruded material [19]. Whereas the mixing screw, equipped with a Maddock section and mixing element of specific geometry, ensures a dispersive type of mixing, in which filler particles and their agglomerates and polymer matrix are exposed to high shear stresses. It yields further improvement of filler particle distribution in samples as well as breaks agglomerated structures of filler particles [19].

The first extrusion process provided melt compounding of the produced LDPE-GnP masterbatches. During this process, only compression screw was used. The obtained material was thereafter pelletized. A second extrusion process was then used to obtain thin films of the nanocomposites with an average thickness of 0.1 mm. The extruder temperatures from the hopper to the die were respectively: 115, 130, 140, and 140 °C. A constant speed of 5 rpm was kept during the process. In total four different LDPE-GnP nanocomposite specimens were prepared, two types manufactured with compression screw (CS) and containing 1 and 5 wt % of GnP and two types manufactured with mixing screw (MS) also containing 1 and 5 wt % of GnP. In addition, pure LDPE specimens were also prepared with the same manufacturing procedure (see Table 2), which act as reference material in the following study.

### 2.3. Characterization Techniques

#### 2.3.1. Scanning Electron Microscopy, SEM

The morphology of the manufactured LDPE-GnP nanocomposites was studied by means of a digital scanning electron microscope Carl Zeiss DSM 940 (Carl Zeiss AG, Oberkochen, Germany). The samples were cooled down in liquid nitrogen and then fractured. The analyzed surfaces were sputtered with gold in vacuum using a Sputter Coater S150B, (approximately 5-nm-thick gold layers, Edwards, Crawley, UK).

#### 2.3.2. Thermogravimetric Analysis, TGA

The filler content and the thermal stability of all specimens were investigated by means of thermogravimetric analyzer, TGA/DSC 3+, Mettler Toledo, Inc., Greifensee, Switzerland. The analyses were carried out under in N_2_ atmosphere with a heating rate of 20 °C/min. Samples of 2–5 mg were used, starting from 30 °C up to 900 °C and kept at 900 °C for 10 min in O_2_ atmosphere.

#### 2.3.3. Differential Scanning Calorimetry, DSC

DSC analysis was used for defining the degree of crystallinity of the studied nanocomposites. The analyzes were performed on 2–5 mg samples using a Mettler Toledo DSC instrument. DSC scans were carried out between −50 °C and 150 °C at a rate of 10 °C/min in N_2_ atmosphere, a heating–cooling–heating cycle was used. The degree of crystallization was calculated by the following equation:

(1) $$\chi_{c}\left({\%}_{crystallinity}\right)=\frac{\Delta H_{m}}{\Delta H_{0}}*100\%$$

where: $\Delta H_{m}$ is the melting enthalpy, and $\Delta H_{0}$ is a theoretical value of the melting enthalpy of 100% crystalline LDPE. The value $\Delta H_{0}$ = 293 J/g was used in a degree of crystallinity calculations [20].

#### 2.3.4. Mechanical Properties

Mechanical properties of the composites were measured by means of Instron 5567 universal testing machine (Instron, Norwood, MA, USA). Tensile tests were performed according standard ISO 37-2. Dog-bone shaped samples were cut parallel and perpendicular to the extrusion direction. All the tests were performed at room temperature and the final results being the average values of five replicated measurements.

#### 2.3.5. Electrical Conductivity

**Figure 2:**
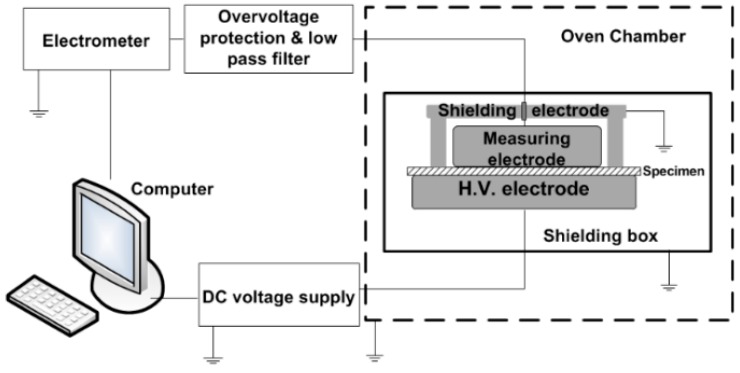
Schematic view of DC conductivity measurement setup.

The used DC conductivity measurement setup is shown in Figure 2. The setup consists of Keithley electrometer 6517B (Keithley Instruments, Solon, OH, USA). It measures the current flowing through the sample. In order to obtain conductivity values for a broad range electric fields, both the electrometer internal voltage supply (up to 1 kV) and a high voltage DC supply (Glassman FJ60R2, 60 kV, 2 mA) were used. To identify the field dependent conductivity, the measurements were performed in a way where the applied voltage was gradually increased in steps of about 4 kV between 8 and 66 kV/mm and the duration of each step was 30 min. All the measurements were performed at room temperature (22 °C). An overvoltage protection together with low pass filters were integrated into the setup in order to prevent possible instrument damage in case of specimen breakdown and for filtering out high frequency noise.

#### 2.3.6. Dielectric Response in Frequency Domain

The dielectric response measurements were performed by IDAX 300 dielectric spectroscopy analyzer (Megger Instruments Ltd., Dover, UK) using the same shielded electrode system as described for DC conductivity measurements. The advantage of using it in the dielectric response measurements is elimination of parasitic capacitances. The measurements were performed in the frequency range of 10^−3^–10^3^ Hz at room temperature (22 °C) at voltage level of 200 V_peak_.

## 3. Results and Discussion

### 3.1. Morphology

**Figure 3:**
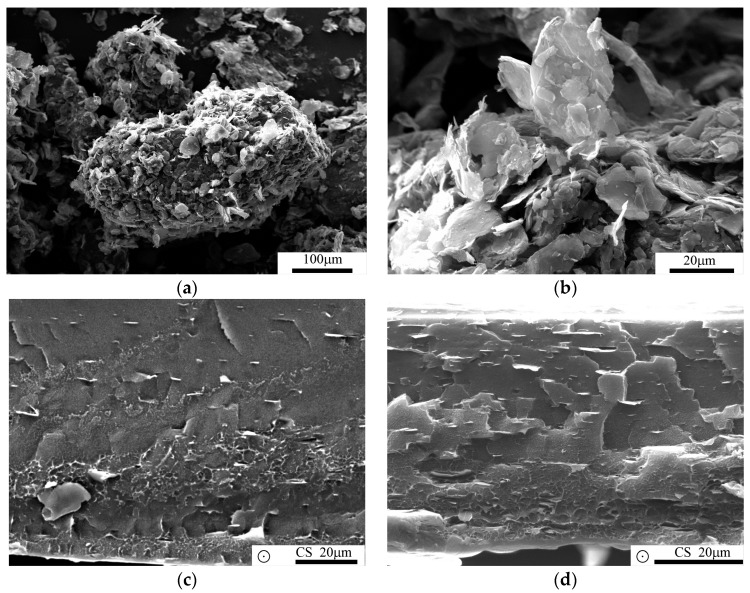

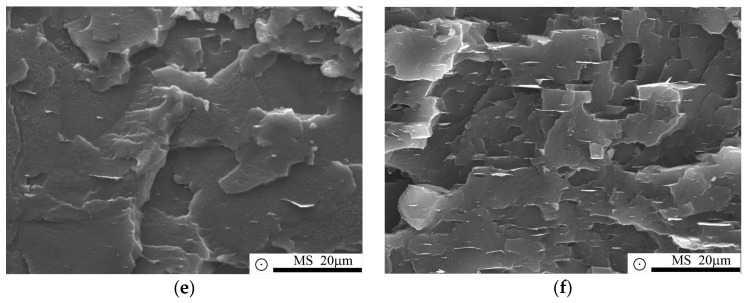
SEM images of LDPE powder coated with GnP nanoplatelets (**a**,**b**); freeze-fractured surfaces of nanocomposites filled with respectively 1% and 5% of GnP produced with a compression screw (**c**,**d**) and with a mixing screw (**e**,**f**). Extrusion direction is indicated by circles.

Figure 3a,b show LDPE particles after the coating process. One can observe a full coverage of their surfaces with GnP nanoplatelets, GnP particles coating LDPE surface is presented on Figure 3b. Figure 3c–f presents morphology of freeze-fractured samples, where the observed surfaces are perpendicular to the extrusion direction. All the figures indicate a strong anisotropy of filler alignment and uniformity of distribution of graphene flakes along polymer flow in the extrusion direction, for both types of the used screws. However, some agglomerated structures can be visible in samples extruded with compression screw (CS), whereas use of mixing screw (MS) allows avoiding this effect. This observation brings us to a conclusion that more efficient filler distribution can be obtained in samples extruded with a mixing screw equipped with a Maddock section and mixing element at its end (see Figure 1).

### 3.2. Thermal Stability

TGA analyses were carried out in order to confirm the content of GnP filler in the nanocomposites. TGA analysis confirmed the filling level as designed, 1.1, 4.62 wt % in case of samples extruded with compression screw and 1.2, 4.98 wt % for samples extruded with mixing screw.

**Figure 4:**
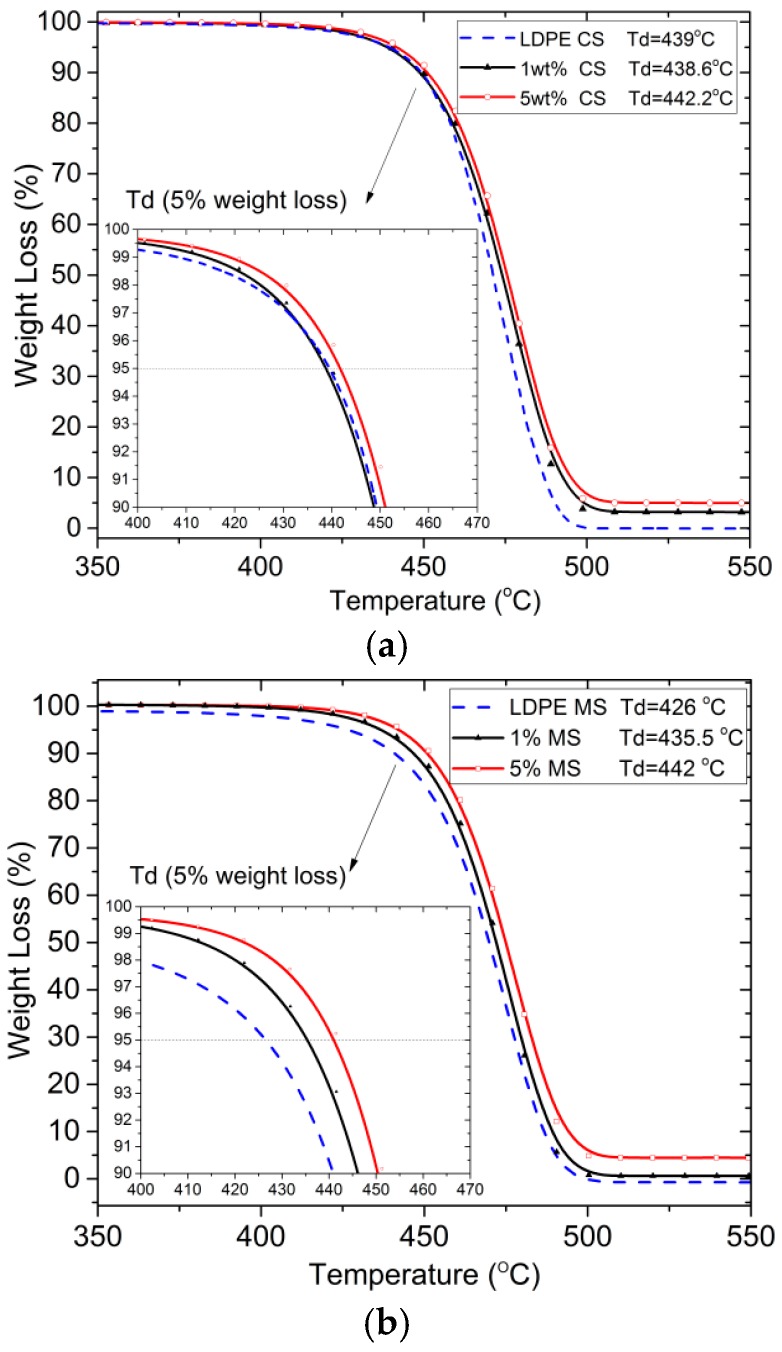
TGA curves measured for specimens produced with a compression screw (**a**) and with a mixing screw (**b**).

The second aspect investigated was the thermal stability *T*_d_ of the nanocomposites, which is extremely important for polymeric materials and often a limiting factor in their manufacturing process and applications. The degradation temperature, defined as the temperature for 5% weight loss, has been extracted from TGA data and is presented in Figure 4.

It can be observed that thermal degradation temperature slightly increased with the increasing filler content. For pure LDPE CS samples *T*_d_ = 439 °C, whereas for CS sample filled with 5 wt % of GnP *T*_d_ = 442 °C. Degradation temperature for MS samples extruded by means of mixing screw LDPE MS *T*_d_ = 426 °C and 5 wt % MS *T*_d_ = 442 °C.

### 3.3. Crystallinity

Incorporation of graphene nanoplatelets into polymer matrix is expected to impact its crystallization degree. As it was earlier observed that nucleation starts around graphite nanoplatelets [15], DSC analyses were performed here in order to investigate this behavior in GnP-LDPE nanocomposites. Table 3 presents crystallization and melting temperatures determined from DSC thermograms as well as crystallization and melting enthalpies. One can see that addition of GnP affects the onset temperature for crystallization *T*_c_. It decreases with increasing filler content. At the same time, the good dispersion of filler particles hinders the growth of LDPE crystallites. The crystallinity degree reduces, starting from 45.9% for pure LDPE CS sample, down to 45.94% for CS sample containing 5 wt % GnP. Crystallinity also reduces for MS samples, starting from 43.8% for pure LDPE MS sample, and going down to 39.8% for MS sample with 5 wt % of GnP. The effect is related to a better dispersion of filler nanoparticles in MS samples.

### 3.4. Mechanical Properties

**Figure 5:**
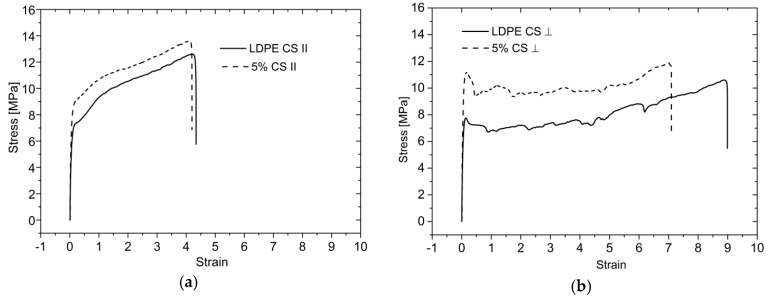

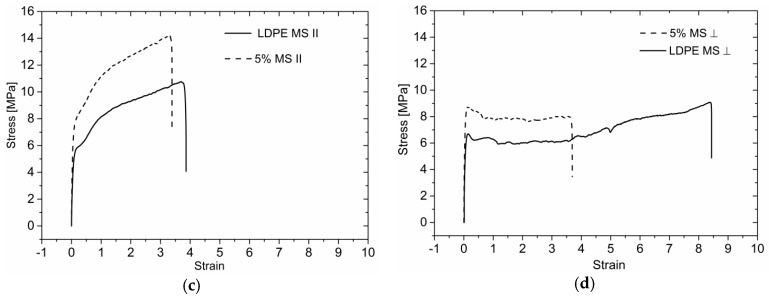
Stress-strain curves measured for reference sample and filled with 5 wt % GnP (**a**) parallel to the extrusion CS; (**b**) perpendicular CS; (**c**) parallel to the extrusion MS; and (**d**) perpendicular to the extrusion.

**Figure 6:**
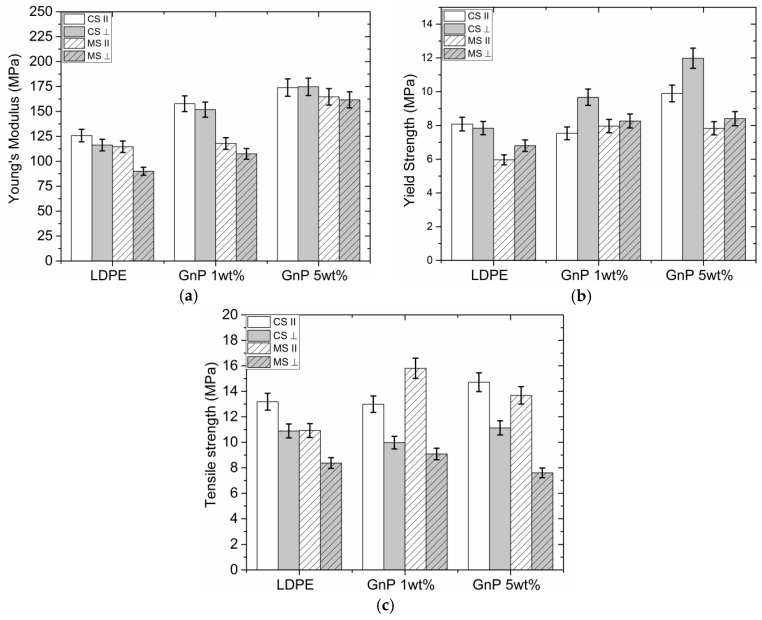
Comparison of Young’s modulus (**a**); Yield strength (**b**,**c**) tensile strength for LDPE-GnP nanocomposites.

Nanocomposites are expected to possess enhanced mechanical properties. Efficient dispersion of filler particles and their adhesion to the base polymer are here prerequisites. Figure 5 presents stress-strain curves for neat LDPE compared with samples filled with 5 wt % GnP content. The extrusion influence can be easily distinguished from stress-strain curves. The shear stress applied during processing leads to strong alignment of GnP flakes along extrusion direction as well as orientation of polymeric chains. One can observe in Figure 5 that maximum strain in both directions (in extrusion direction and perpendicular) decreases with increasing filler content and tensile strength increases for samples with a filler (see Figure 6). It is visible for samples manufactured by means of mixing screw, better dispersion of GnP leads to reduced polymer chain mobility, which reduces maximum strain especially in transverse direction.

Figure 6a presents results of Young’s modulus measurements for samples cut parallel and perpendicular to the extrusion direction. Figure 6b represents the tensile strength of the measured nanocomposites and, thereafter, Figure 6c shows yield strength as follows. As observed in Figure 6a, Young’s modulus increased for samples in both directions, the MS samples exhibit a higher Young’s modulus with 79% increase for samples cut perpendicular to the extrusion direction and 43% for samples cut in parallel. Young’s modulus of CS samples increased by 38% for samples cut parallel to the extrusion direction and by 50% for samples cut perpendicular. By this comparison, one can see a substantial influence of the strong anisotropy and alignment of graphene particles as well as the flow direction during the extrusion on the mechanical properties. Figure 6b shows yield strength which also increased in all samples with the filler. Figure 6c shows the results of tensile strength tests, which stayed at the same level for all samples. The increase in mechanical properties is not significant, probably due to lack of compatibility between GnP and LDPE matrix. Therefore, surface modification and improvement of the interface is essential to increase stress transfer through GnP particles. Moreover, wrinkled and not fully exfoliated GnP surface could also have influenced the overall composite stiffness [11,14].

### 3.5. Electrical Properties

#### 3.5.1. DC Conductivity

**Figure 7:**
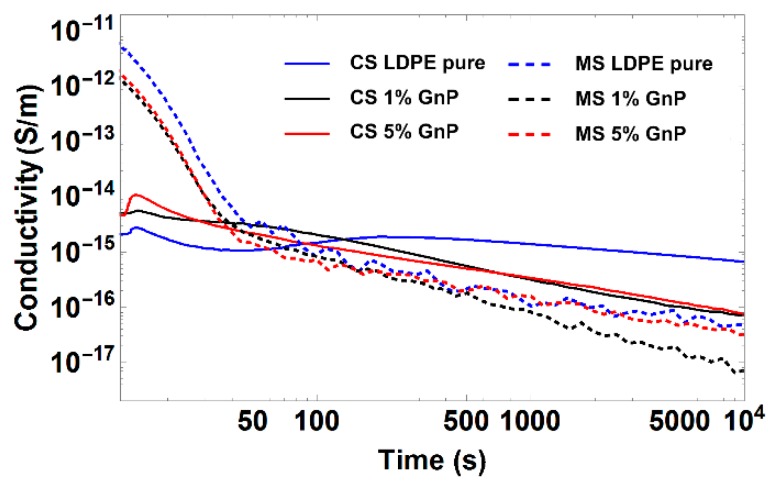
DC conductivity of produced nanocomposites, CS—solid line and MS—dashed line measured at a constant field 10 kV/mm.

Figure 7 presents results of time dependence of charging current measurements, representing electrical conductivity of the investigated nanocomposites. One can observe that samples with 1 and 5 wt % show lower DC conductivity as compared to the reference pure LDPE. It can be assumed that GnP particles aligned perpendicular to the electric field act as charge trapping sites, reducing transport of electric charges through the material this way. This effect is most significant at the lower filler concentration (1 wt %) and at a relatively low electric field. It has been already observed in several studies that introducing filler particles into LDPE matrix can significantly reduce its conductivity, when for example using nanofiller particles of MgO, Al_2_O_3_, SiO_2_, or ZnO [21,22,23,24,25]. It has also been confirmed in [26] that introducing low concentrations of GnP into polyethylene reduces effective conductivity of LDPE-GnP composite. A similar behavior was reported when adding graphene oxide into polydimethylsiloxane (PDMS) matrix [27].

**Figure 8:**
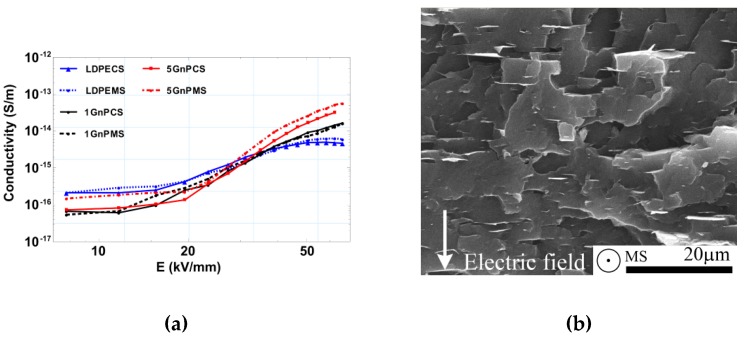
Field dependence of conductivity (30 min values) at 22 °C for pure LDPE and its GnP nanocomposites (**a**), direction of electric field (**b**).

In Figure 8, field dependences of conductivity measured for all the investigated samples are compared. A nonlinear behavior starts to dominate this property at a field strength of about 20 kV/mm. A clear crossover effect is also observed, where at low field conductivity of the filled nanocomposites turns into higher conductivity of pure LDPE CS and LDPE at higher field strength, being strongest exhibited in MS samples containing 5 wt % of GnP. There also seems to exist a tendency for the conduction to saturate at fields about 40 kV/mm for pure LDPE samples and above this level for the nanocomposites. A higher DC conductivity obtained for a 5 wt % MS GnP sample is a result of better dispersion of graphene nanoplatelets, as better GnP dispersion leads to higher DC conductivity. What is more, this behavior will correspond to the crystallinity of LDPE matrix. Samples processed with mixing screw possess lower crystallinity and this strictly corresponds to the lower tendency of charge accumulation on crystalline lamellas in an LDPE matrix.

#### 3.5.2. Dielectric Response

**Figure 9:**
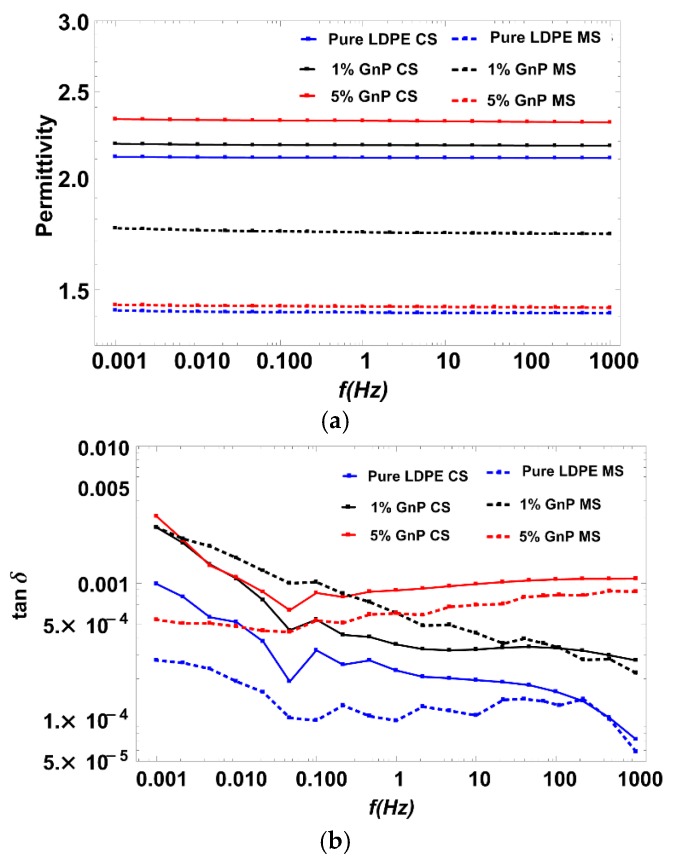
Dielectric response of CS vs. MS (**a**) dielectric constant and (**b**) dielectric loss.

Figure 9 presents dielectric variations of dielectric permittivity and dissipation factor for the investigated samples. Increases of both dielectric permittivity and losses are observed with the increasing level of GnP filler content for CS samples. In contrast, MS samples exhibit a decrease of the dielectric permittivity and the dielectric losses as compared to CS samples. This effect requires more detailed investigations as the lower permittivity and losses are beneficial in electrical insulation applications.

## 4. Conclusions

It is proved by the results presented here that precoating method combined with extrusion by means of a mixing screw allows the manufacture nanocomposites of LDPE filled with GnP nanoplatelets that are characterized by good filler dispersion and decreased concentration of agglomerates. The extruded nanocomposites show strong anisotropy in filler distribution, which enhance their mechanical properties and yield non-linear field dependent behavior of electrical conductivity.

A lower conductivity, as compared to pure LDPE, is observed at electric field levels below 10 kV/mm, while at higher fields the non-linear behavior starts dominating. It is believed that interfacial regions between LDPE and filler particles play a dominant role here by controlling space charge movement in the material bulk. On the other hand, the type of extrusion screw used for sample manufacturing affects the dielectric response characteristics of the nanocomposites differently. Increases of dielectric permittivity and dissipation factor with the increased level of GnP filler are found for samples produced with a compression screw, whereas the samples produced with a mixing screw show an opposite effect. Presented findings show that graphene nanoplatelets can be used in future applications, in which the non-linear behavior of electric conductivity is advantageous, as for example in electric field grading elements of high voltage direct current (HVDC) cable terminations and joints.

## Figures and Tables

**Table 1:** Basic characteristics of the low-density polyethylene and GnP nanoplatelets as a filler.

Graphene nanoplatelets: xGnP M5		Low density polyethylene	
Surface Area (m^2^/g)	120–160	*M*_w_	91,641
Average diameter—*d*_ave_ (µm)	25	*M*_w_/*M*_n_	7.552
Thickness (nm)	6–8	*T*_m_ (°C)	110.62
Density *ρ* (g·cm^−3^)	2.2	*T*_c_ (°C)	94.09

**Table 2:** Produced samples.

	Screw type	Filler content (wt %)	Name of sample
Samples	CS	1	1 wt %_CS
		5	5 wt %_CS
		Pure LDPE	LDPE CS
	MS	1	1 wt %_MS
		5	1 wt %_MS
		Pure LDPE	LDPE MS

**Table 3:** Basic characteristics of the low density polyethylene and GnP nanoplatelets as a filler.

Sample	*T*_c_ (°C) Crystallization temperature	Δ*H*_c_ (J/g) Crystallization enthalpy	*T*_m_ (°C) Melting temperature	Δ*H*_m_ (J/g) Melting enthalpy	χ_c_ (%) Crystallinity degree
LDPE-CS	98.15	132.52	110.62	134.52	45.91
LDPE_GnP_1 wt % CS	103.61	124.31	110.84	131.70	44.95
LDPE_GnP_5 wt % CS	103.82	126.47	107.98	134.60	45.94
LDPE-MS	99.59	124.37	107.79	128.39	43.82
LDPE_GnP_1 wt % MS	98.60	118.03	106.91	123.34	42.10
LDPE_GnP_5 wt % MS	99.35	117.63	106.52	116.55	39.78
